# Life Assurance
†On the Medical Estimate of Life for Life Assurance. By Stephen H. Ward, M.D.Lond. pp. 71. London: 1857.


**Published:** 1857-10

**Authors:** 


### LIFE ASSURANCE.†

Dr. S. Ward publishes a useful little handbook for Medical Referees to assurance offices. The different elements which enter into the medical estimate of life are reviewed ; viz., Sex, Age ; Married or Single State ; Personal Peculiarities ; Habits ; Occupation ; Residence ; Family History ; Previous Illnesses or Accidents ; Vaccination ; Present Health ; and Peculiarities

---

† On the Medical Estimate of Life for Life Assurance. By Stephen H. Ward, M.D.Lond. pp. 71. London: 1857.

affecting the Female. The temperate friends of the pipe will read with satisfaction Dr. Ward's opinion that there is no evidence whatever to show that this practice (smoking tobacco), "when had recourse to in moderation, and not complicated with spirit-drinking, at all tends to shorten the duration of life. Addiction to it in great excess may doubtless induce dyspepsia, nervous affections, possibly paralysis, certainly delirium tremens."

